# Climate change: a critical time for respiratory health

**DOI:** 10.36416/1806-3756/e20250237

**Published:** 2026-05-22

**Authors:** José Miguel Chatkin, Rafael Futoshi Mizutani, Ubiratan Paula Santos

**Affiliations:** 1. Divisão de Pneumologia, Hospital São Lucas, Pontifícia Universidade Católica do Rio Grande do Sul - PUCRS - Porto Alegre (RS) Brasil.; 2. Divisão de Pneumologia, Instituto do Coração - InCor - Hospital das Clínicas, Faculdade de Medicina, Universidade de São Paulo - HCFMUSP - São Paulo (SP) Brasil.

**Keywords:** Climate change, Air pollution, Global warming, Global health, Extreme weather

## Abstract

Climate change has consistently been shown to result from human activity. The rise in average global temperature, driven by greenhouse gas emissions, has increased the frequency of extreme weather events such as floods, droughts, hurricanes, wildfires, and heat waves, as well as increasing airborne allergens and air pollution levels. These consequences have direct and indirect impacts on human health, particularly affecting individuals with cardiorespiratory disease. This review analyzed the main mechanisms whereby climate change impacts human health, with a focus on respiratory effects. Urgent mitigation efforts to reduce carbon emissions and prepare for extreme weather events are needed and must involve coordinated efforts across society. Physicians play a crucial role in communicating to patients how climate change affects health and in encouraging contributions to mitigation efforts by everyone.

## INTRODUCTION

**Figure 1:**
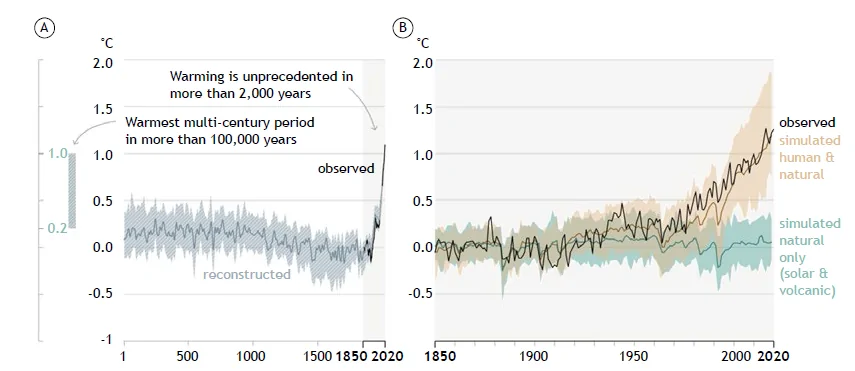
History of global temperature change and causes of recent warming. In A, changes in global surface temperature reconstructed from paleoclimate archives (solid gray line, 1-2000) and from direct observations (solid black line,1850-2020), both relative to 1850-1900 and decanally averaged. In B, changes in global surface temperature over the past 170 years (black line) relative to 1850-1900 and annually averaged.^2^

**Figure 2:**
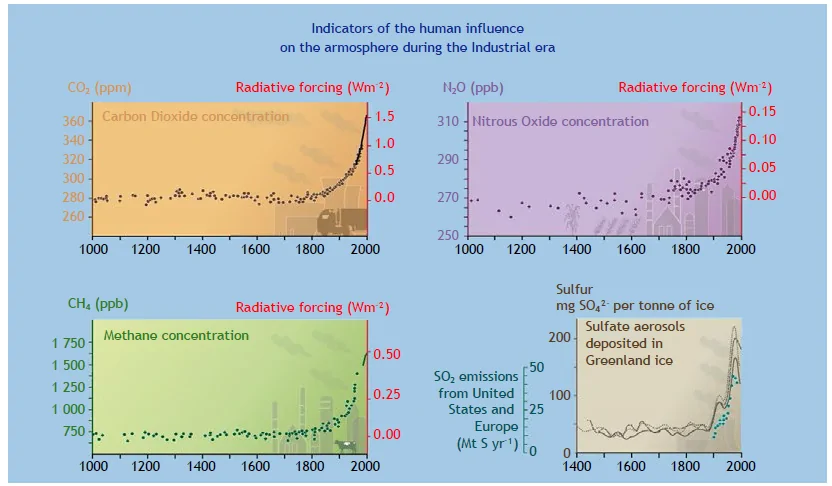
Indicators of the human influence on the atmosphere during the industrial era.^2^

There is clear evidence that climate change resulting from human activity is associated with an increase in extreme events and changes in ecosystems, as well as direct and indirect effects on human health. From the Second Assessment Report of the Intergovernmental Panel on Climate Change (AR2, 1995)^1^ to the Sixth Assessment Report of the Intergovernmental Panel on Climate Change,AR6^2^ studies have consistently demonstrated the role of human activity in increasing pollutant emissions and their implications for rising temperatures since 1850 (Figures 1 and 2).^2^ ^,^ ^3^

The atmospheric concentration of greenhouse gases (GHGs) has significantly increased since the first Industrial Revolution in the mid-18th century, particularly in the last 80 years. This rise in mainly due to the sharp increase in fossil fuel consumption driven by growing vehicle traffic and energy demand, combined with intense deforestation of natural landscapes to make way for agriculture and livestock activities.^2^

The increase in average global temperature is mainly due to the release of GHGs such as CO_2_, CH_4_, and N_2_O. These gases persist for hundreds of years, accumulate in the troposphere, and absorb solar radiation, causing a long-lasting rise in global temperatures. Ozone layer depletion caused by the release of halogenated hydrocarbons (including hydrofluorocarbons, chlorofluorocarbons, and perfluorinated compounds) contributes to global warming by allowing more solar radiation to reach the surface of the Earth. As a result, the world is experiencing rising sea levels and an increase in heat waves and extreme weather events such as droughts, storms, floods, and significant El Niño events due to the overheating of the equatorial Pacific Ocean waters.^2^

Warming temperatures and prolonged droughts contribute to large-scale fires. Many sources of GHG emissions, such as burning fossil fuels, biomass burning, and industrial activities, are also main contributors to the emission of pollutants such as particulate matter (PM), gases, and volatile organic compounds that adversely affect human health.^4^

Quantifying the direct impact of climate change on human morbidity and mortality is challenging. The Sixth Assessment Report of the Intergovernmental Panel on Climate Change AR6^5^ estimated that 3.3-3.6 billion people live in areas that are highly vulnerable to climate change. Heat-related mortality alone is estimated at 300,000 to 500,000 excess deaths annually.^6^ ^,^ ^7^ The 2024 World Economic Forum Report estimated that, by 2050, climate change will have caused an additional 14.5 million deaths and $12.5 trillion U.S. dollars in economic losses worldwide.^8^

Climate change poses many risks to human health. Some health impacts are evident even in countries with higher human development indices (HDIs), such as the United States, Canada, and European countries. However, health impacts are more common and more devastating in developing countries, further exacerbating social inequalities.^9^ ^-^ ^11^ This disparity is intensified by a lack of universal health care systems, limited financial resources, and a lower socioeconomic status. According to the Sixth Assessment Report of the Intergovernmental Panel on Climate Change AR6, South and Central America are highly exposed and vulnerable to the effects of climate change.^2^

The combined effects of pollutants, global warming, and land degradation lead to changes in flora, increasing pollen production with greater allergenic potential, which is linked to a higher incidence of rhinitis and asthma. They also raise the risk of vector-borne diseases such as malaria and dengue fever through shifts in vector distribution-mosquitoes thrive in warmer, more humid conditions-and increase human-vector contact due to deforestation. Floods heighten the risk of waterborne diseases such as cholera, dysentery, leptospirosis, and protozoan infections. Disruptions in food production caused by prolonged droughts or excessive rainfall contribute to increased malnutrition and food insecurity.^12^ ^,^ ^13^

Heat waves combined with humidity can trigger severe asthma symptoms much faster than can cold waves, which affect individuals after a lag period.^14^ Heat waves are also associated with an increased risk of exacerbations and death in individuals with COPD,^9^ as well as increased hospitalizations and emergency room visits for asthma. Heat waves negatively impact lung function and increase the risk of infection in COPD patients and healthy individuals.^15^ ^,^ ^16^ The elderly and those with cardiorespiratory diseases are the most vulnerable groups, requiring more emergency room visits and hospitalizations, as well as facing a higher risk of mortality.^17^ Children also require increased pediatric care.^18^ ^,^ ^19^

Physicians play a vital role in addressing climate change by raising awareness of its direct and indirect health impacts. They serve as key advocates in educating the public on individual and collective measures to mitigate its consequences. This approach parallels the successful tobacco control campaigns of recent decades, which depended on coordinated governmental efforts to implement policies designed to reduce tobacco consumption (Chart 1).^20^

**Chart 1:** Impact of climate change on human health.

• Human activities are responsible for an average increase of 1.5°C in the earth’s global temperature in comparison with the preindustrial period. This increase could reach 2°C or more by 2050. • In some regions of the earth, the increase is two to three times higher than the global average. • The main greenhouse gases are CO_2_, CH_4_ and N_2_O. • The reduction of the ozone layer in the stratosphere increases the intensity of the solar radiation that reaches the earth’s surface, where, adsorbed by greenhouse gases, it raises the temperature, as well as increasing the generation and concentration of ozone on the earth’s surface through photochemical reactions. • The warming caused to date could last for centuries or even millennia, with an impact on the climate, sea levels, and agriculture. • The number of deaths per year is estimated at 250,000 from 2030 onwards. • The main impacts on health are due to the increased frequency and intensity of heat waves, increased humidity, and increased concentrations of pollutants, factors that are associated with increased morbidity and mortality from respiratory and cardiovascular diseases. • The impacts on the lives and physical and mental health of people affected by extreme events, with temporary or permanent displacement of populations, are not negligible.

## CLIMATE CHANGE AND ALLERGENS

Higher atmospheric carbon dioxide concentrations and increased temperatures act synergistically to boost pollen production and extend the pollen season, resulting in an increase of up to 40% in airborne pollen.^21^ Air pollution also alters the chemical composition of pollen particles, potentially increasing their allergenicity. When sensitive individuals are exposed to allergens and air pollutants, allergic reactions tend to be more severe.^22^

Thunderstorm asthma events are also becoming more common. These occur when extreme weather events (e.g., storms and hurricanes) cause dramatic spikes in airborne pollen levels, triggering asthma attacks in susceptible individuals. During such events, strong winds increase pollen release into the atmosphere, and, upon contact with water, pollen ruptures through osmotic shock, releasing particles that have increased allergenic potential and that can cause asthma attacks. These events affect many individuals simultaneously, overwhelming local emergency care and intensive care services.^23^

## HEAT-RELATED ILLNESS

Although exposure to cold weather has a greater impact on mortality, heat-related mortality has risen in the last decades because of global warming, with an estimated 489,000 annual deaths worldwide from 2000 to 2019.^7^ Deaths from heat may result directly from inability to dissipate excessive heat (heat exhaustion and heatstroke) or indirectly as a risk factor for cardiovascular, respiratory, gastrointestinal, and kidney diseases.^7^

Classic heatstroke is a severe condition caused by failure to dissipate excessive body heat, mainly affecting children and vulnerable elderly individuals during heat waves. In contrast, exertional heatstroke affects athletes, firefighters, soldiers, agricultural workers, outdoor workers, or anyone undergoing intense physical activity in a hot environment. In both cases, heatstroke leads to hyperthermia, neurological impairment, and systemic inflammatory response, which can result in multiorgan dysfunction and death.^24^ Heatstroke epidemics are expected to become more common because of the aging population, increased social isolation among the elderly, and more frequent, longer, and intense heat waves.^25^

Higher temperatures also increase ground-level ozone, an air pollutant linked to more medical outpatient visits and hospitalizations caused by loss of asthma control, as well as reduced lung function caused by inflammation of bronchial epithelial cells, leading to airway hyperresponsiveness, exacerbation, and death in COPD patients. Warmer temperatures combined with higher ozone exposure impair the fibrinolytic pathway, increasing cardiovascular events such as myocardial infarction and stroke.^26^ ^-^ ^28^

## AIR POLLUTION AND CARDIORESPIRATORY DISEASES

Air pollution is the leading global risk factor for disease burden, accounting for approximately 8% of the total disability-adjusted life years and surpassing high systolic blood pressure and smoking.^29^ Air pollution was responsible for over eight million deaths globally in 2021.^29^ The health effects of air pollution and climate change disproportionately affect underserved communities. Children are especially susceptible because their defenses are still developing. Elderly people with preexisting conditions are also more affected by climate change. People facing food insecurity and economic hardship are more likely to be exposed to environmental risks and suffer greater impacts.^2^

**Figure 3:**
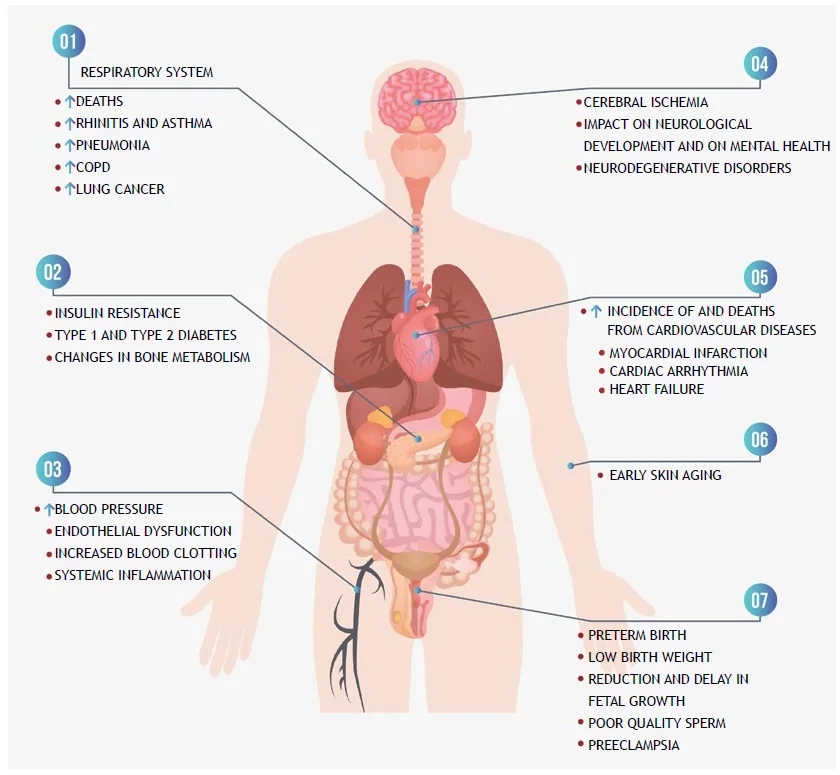
Representation of health effects associated with air pollution.^30^

Exposure to environmental air pollutants can cause acute effects by exacerbating chronic diseases and triggering acute cardiopulmonary and infectious events. Chronic exposure is linked to increased incidences of asthma, COPD, lung cancer, neurological disease, metabolic disease, cardiovascular disease, and infertility (Figure 3).^30^

Climate change increases PM; the greater the increase, the higher the risk of cardiopulmonary illness and reduced life expectancy.^30^ Tropospheric ozone concentrations also rise, triggering asthma attacks, allergy symptoms, and increased COPD mortality.^31^ A longer drought season as a result of climate change causes more frequent and more intense wildfires. 

Fine PM (PM_2.5_) and ultrafine (PM_0.1_) particles penetrate deep into the lungs, harming respiratory health and heart function. These particles cause oxidative stress, inflammation, autonomic nervous system imbalance, and toxin transmission into the bloodstream, leading to hypertension, heart failure, stroke, high cholesterol, arrhythmias, and insulin resistance.^30^

New cases of childhood asthma caused by air pollution are estimated at 4 million annually, with 150,000 cases occurring in tropical Latin America.^32^ Research shows that children living in areas with high ozone and nitrogen oxides (NO and NO_2_) levels are more likely to develop asthma.^33^ ^,^ ^34^ Climate change poses a particular threat to these patients. Asthma patients have weakened antioxidant defenses, making them especially vulnerable to ozone and PM exposure. 

**Figure 4:**
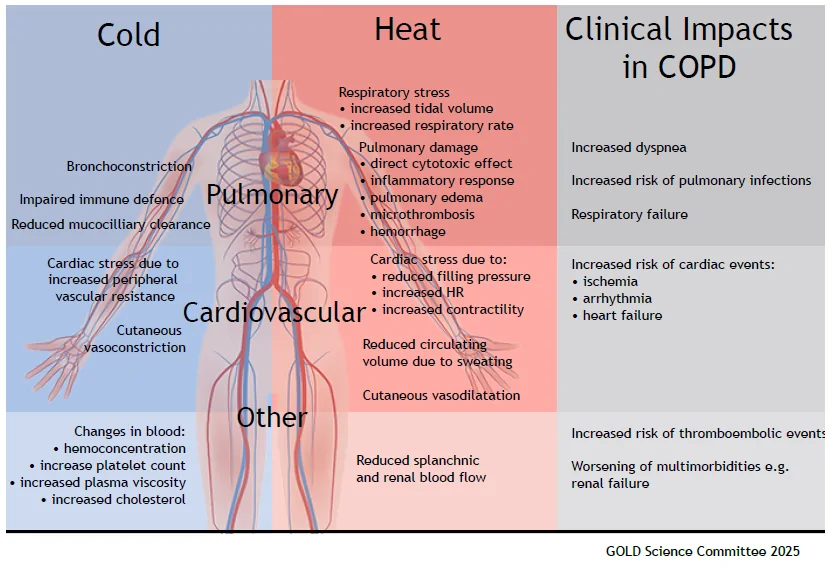
Acute effects of temperature change on COPD patients.^35^

Patients with COPD face increased mortality risks from extreme heat and cold exposure. Respiratory and cardiovascular events are the leading causes of death in this population. Warmer temperatures increase the risk of death by up to 25%, whereas extreme cold can raise it by approximately 20% during winter.^35^ Mortality in patients with COPD notably rises 2-3 days after severe temperature drops. Generally, cold weather presents a higher mortality risk from COPD than does heat because of increased cardiac stress, infection-related exacerbations, and inflammation with airway epithelial damage. Cold-induced bronchoconstriction may also contribute. Heat waves have immediate effects, whereas cold-related deaths typically lag several days. Therefore, COPD patients should be informed about these risks to mitigate their impact. (Figure 4).^35^

## WILDFIRES AND AIR POLLUTION: SHORT- AND LONG-TERM EFFECTS

**Figure 5:**
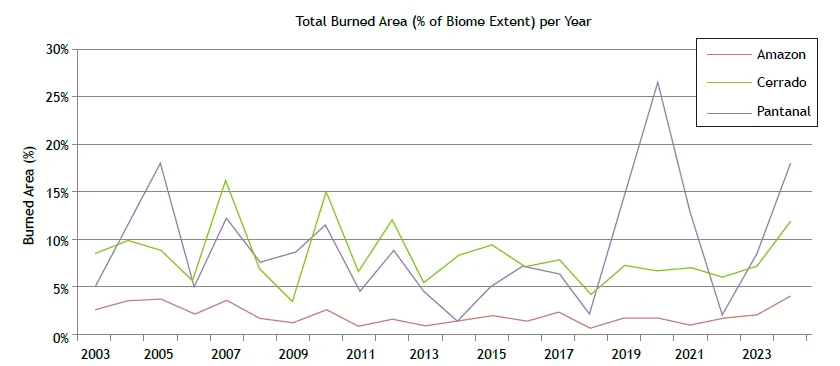
Total burned area (in percentage of biome extent) per year since 2003.^36^

In recent years, large areas of Brazil, Canada, the United States, and Australia, as well as many European countries, experienced very poor air quality and smoke inhalation from difficult-to-control wildfires. A series of record-breaking wildfires occurred in several regions in Brazil, such as the Pantanal biome in 2020, with 26.4% of the total biome extension being burned that year, and 2024 (18.1%); the *Cerrado* in 2024 (11.9%); and the Amazon rainforest in 2024 (3.9%; Figure 5).^36^ Not only did these fires threaten the residents but also affected distant regions, given that PM is easily carried by atmospheric circulation. Warmer temperatures, lower humidity, longer dry seasons, and shifting wind patterns are becoming more common because of climate change, creating conditions that facilitate the ignition of longer lasting and larger wildfires. Carbon dioxide emission and degraded land caused by these wildfires further exacerbate climate change, creating a vicious cycle.^37^

In communities near wildfire areas, there have been reports of increased emergency room visits, especially for respiratory diseases such as asthma and COPD, as well as cardiovascular diseases such as stroke and myocardial infarction.^38^ Rising hospitalization and mortality put immense pressure on the health care system during and after the wildfires. Certain segments of the population are particularly vulnerable to smoke-related health risks, such as the elderly, smokers, pregnant women, and firefighters. The effects of wildfire smoke can range from eye and respiratory irritation to more severe conditions, including reduced lung function, bronchitis, asthma exacerbations, acute cardiovascular diseases, and premature death.^39^ Children whose mothers were exposed to wildfire smoke during pregnancy are at a higher risk of preterm birth, low birthweight, neurodevelopmental disorders, and asthma.^40^ ^,^ ^41^

## MEASURES TO MITIGATE CLIMATE CHANGE

Mitigating climate change is an urgent task that requires coordinated efforts across society. Governments must pursue assertive agendas to reduce reliance on fossil fuels, promote green energy infrastructure, support the development of clean and/or energy-efficient technology, prepare for extreme weather events and their consequences, and protect natural landscapes from deforestation. Corporations should aim for net-zero emissions and prioritize partnerships with other environmentally friendly businesses. Civil society should also play a role by reducing individual energy consumption and actively choosing activities and products with lower carbon footprint, such as using public transportation and walking/cycling instead of driving. 

Efforts to improve air quality align with broader climate change mitigation policies but focus on reducing pollutants such as PM, ground-level ozone, and nitrogen oxides. Reducing fossil fuel consumption for energy production, improving public transportation, rethinking urban planning, regulating industrial emissions, and limiting deliberate landscape and agricultural fires are examples of policies that are aimed at reducing air pollutants and carbon emissions. 

There is clear evidence that improving air quality can restore public health. In the state of California, USA, researchers have reported improvements in lung development in children as a result of improved air quality.^42^ In the USA, data from metropolitan areas showed that a reduction in PM_2.5_ led to a seven-month increase in life expectancy between the 1980s and the 1990s, accounting for 15% of the total life expectancy gain during that period.^43^ In China, a strict air pollution control program focusing on power generation, industry, household fuel use, and transportation has resulted in marked improvements in air quality. In 2022, Beijing recorded PM_2.5_ levels below the WHO target for the first time.^44^

Urban planning, including the expansion of green and blue space, should be actively incorporated into city master plans. Green infrastructure includes parks, gardens, urban farms, and forested areas, whereas blue infrastructure includes rivers, lakes, canals, and other bodies of water. This approach is aimed at integrating nature into urban life, offering environmental, social, and economic benefits. During heat waves, green areas, parks, and public spaces near bodies of water are cooler than paved urban areas, potentially mitigating heat-related illnesses. Not only does urban greening have a local cooling effect but also reduces ground-level nitrous oxide levels without affecting local ozone concentrations.^45^

Sustainable urban planning should also focus on promoting public transportation and cycling infrastructure, reducing waste, increasing recycling, optimizing buildings to lower energy consumption, producing sustainable energy locally (e.g., solar panels and wind turbines), and shortening commuting distances by encouraging job opportunities near residential areas. These measures can help reduce carbon footprints and improve public well-being.^46^

Brazil is in a unique position to lead global climate change mitigation efforts. The United Nations climate summit will be held for the first time in the Amazon under Brazilian leadership during the 2025 United Nations Climate Change COP30 (Conference in the city of Belém. This is a tremendous opportunity to draw the world’s attention to the importance of the Amazon rainforest not only as a biodiversity reservoir but also as a crucial regulator of weather patterns and carbon absorber. Its preservation is vital not only to Brazil but also to the entire planet. 

## KNOWLEDGE GAPS AND FUTURE RESEARCH

Although data on the health effects of climate change are increasing, there remains significant research to be done in several areas. Because most research efforts are primarily led by high-income countries,^47^ there is a fundamental lack of representation from leading authors in low- and middle-income countries. Such countries may experience unique consequences and are more likely to focus research on vulnerable populations, such as Indigenous peoples and isolated communities. Climate change mitigation strategies will be implemented differently across locations, depending on local budgets, access to technology, and specific contextual factors. As a result, their impact on health will also vary. Therefore, it is essential to include diverse perspectives in research efforts. 

Regarding the direct and indirect effects on lung health, there is a lack of research on long-term outcomes.^47^ ^,^ ^48^ Most existing studies focus on short-term respiratory effects of wildfires and extreme weather events. Additionally, the combined effects of temperature, air pollution, and allergens are still poorly understood.^48^

Understanding the multiple pathways through which climate change and human health interact may lead to new and unexpected solutions. Interdisciplinary collaboration among physicians, epidemiologists, meteorologists, urban planners, and policymakers is crucial for developing effective strategies to protect populations from the health impacts of climate change. 

## FINAL CONSIDERATIONS

The emission of GHGs and air pollution resulting from an increasing urbanization, followed by rising fossil fuel consumption, energy demand, and biomass burning (common in agricultural practices), combined with deforestation and reduction of green spaces, as well as the verticalization of urban areas in many countries, including Brazil, are the primary drivers of climate change. The effects of climate change on human health have been well documented since the late 20th century. This creates a concerning scenario, as the impacts on human morbidity, mortality, and socioeconomic inequality continue to increase, yet the countries with the largest carbon footprint have failed to take sufficient action to reduce emissions. 

To mitigate the effects of climate change, the only viable solution is to reduce pollutant emissions, halt deforestation, expand green areas, conserve water, adopt building practices that require less energy consumption, generate energy from cleaner sources, and transform current agricultural practices for food production. Simultaneously, urgent mitigation policies are necessary to reduce the impacts of climate change that are already underway due to human activities and to support the most vulnerable populations with access to housing, water, sanitation, food, and employment. These policies must persist over long periods, given that the impacts of climate change on air, land, and oceans are long-lasting. 
